# Development and validation of an interpretable machine learning model for predicting the risk of non-cardiac surgery postoperative heart failure: a multicenter study

**DOI:** 10.3389/fmed.2025.1666885

**Published:** 2025-12-11

**Authors:** Qing Li, Zizhou Liu, Kunlun He, Yan Zhuang, Junyan Zhang, Bing Wei, Hebin Che, Bo Zhang, Liandi Jiu, Jiayue Li, Xinyu Song, Wei Dong

**Affiliations:** 1 Department of Medicine, South China University of Technology, Guangzhou, China; 2 Department of Cardiology, The Sixth Medical Centre, Chinese PLA General Hospital, Beijing, China; 3 Medical Innovation Research Department, The First Medical Centre, Chinese PLA General Hospital, Beijing, China; 4 Department of Medical Informatics, The Sixth Medical Center, Chinese PLA General Hospital, Beijing, China; 5 Artificial Intelligence Institute, Digital Health China Technologies Co. Ltd, Beijing, China

**Keywords:** non-cardiac surgery, heart failure, machine learning, risk prediction model, postoperative, clinical decision support

## Abstract

**Background:**

This study developed a machine learning model to predict postoperative heart failure (HF) risk in non-cardiac surgery patients.

**Methods:**

Using data from 489 patients (109 HF cases, 380 controls), the dataset was split 8:2 into training and testing sets, with under-sampling for class imbalance. Eight algorithms were evaluated, with random forest (RF) performing best.

**Results:**

The RF model achieved AUROCs of 0.919 (training) and 0.923 (testing), validated externally (AUC = 0.878). SHAP analysis identified key predictors: age, neutrophil-to-lymphocyte ratio, blood glucose, INR, pulse and serum creatinine (positively associated); serum albumin, MCHC, eGFR and diastolic blood pressure (negatively associated). A web-based tool was developed for clinical use.

**Conclusion:**

The model integrates 10 clinical variables reflecting age, inflammation, renal dysfunction, and hemodynamic instability, enabling preoperative risk stratification and guiding targeted interventions to improve perioperative outcomes.

## Graphical abstract

**Figure:**
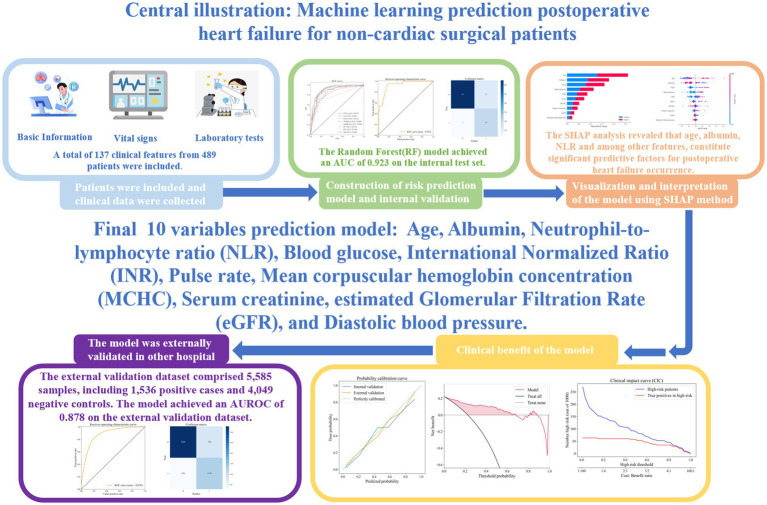

## 1 Introduction

Each year, over 300 million major surgical procedures are performed globally—approximately 5% of the population—with nearly 85% classified as non-cardiac surgeries (1). In the United States, more than 1 million patients are hospitalized annually for such operations (2). Perioperative heart failure occurs in approximately 4.9% of cases and is associated with poor outcomes. Therefore, early and accurate prediction of postoperative heart failure is crucial for optimizing perioperative care and improving prognosis.

In 2024, the American College of Cardiology and the American Heart Association released updated guidelines emphasizing the importance of preoperative evaluation and risk stratification to reduce perioperative mortality in non-cardiac surgery patients (3). Risk assessment tools such as the Revised Cardiac Risk Index (RCRI) and the American University of Beirut Heart and Arterial Stiffness Score (AUB-HAS2) have been developed (4, 5), but both have limitations. For instance, the RCRI categorizes all intraperitoneal and intrathoracic surgeries as high risk, failing to reflect advances in minimally invasive procedures (4). The AUB-HAS2 index incorporates symptoms such as angina and dyspnea as core criteria. While clinically important, these subjective measures may be underreported or masked in elderly or frail patients with limited mobility, thereby undermining the objectivity and consistency of risk stratification (5). Moreover, most existing models are based on historical datasets and outdated surgical practices, limiting their applicability to contemporary patient populations and modern perioperative care settings.

With the rising prevalence of heart failure risk factors such as hypertension and diabetes (6), the incidence of perioperative heart failure is expected to increase. However, most current tools focus on major adverse cardiac events and often overlook heart failure as an independent endpoint. Current research primarily focuses on predicting myocardial infarction or sudden death risk in patients with known chronic heart failure, while risk prediction for patients without pre-existing heart failure has received comparatively less attention.

The rapid advancement of artificial intelligence presents new opportunities for personalized risk stratification and precision medicine (7–9). However, the application of machine learning methods in predicting perioperative heart failure has not yet received sufficient research attention. Machine learning-based risk prediction models may overcome the shortcomings of traditional approaches, particularly their limited ability to incorporate a broad range of relevant clinical variables. Utilizing multicenter data from patients having non-cardiac surgery, this study integrates vital signs and laboratory indicators extracted from electronic medical records and applies eight widely used machine learning algorithms to develop and externally validate an interpretable model for postoperative heart failure prediction. The objective is to establish a reliable clinical tool for the early identification of high-risk patients and the optimization of preoperative management strategies.

## 2 Materials and methods

### 2.1 Research subject

**Figure 1:**
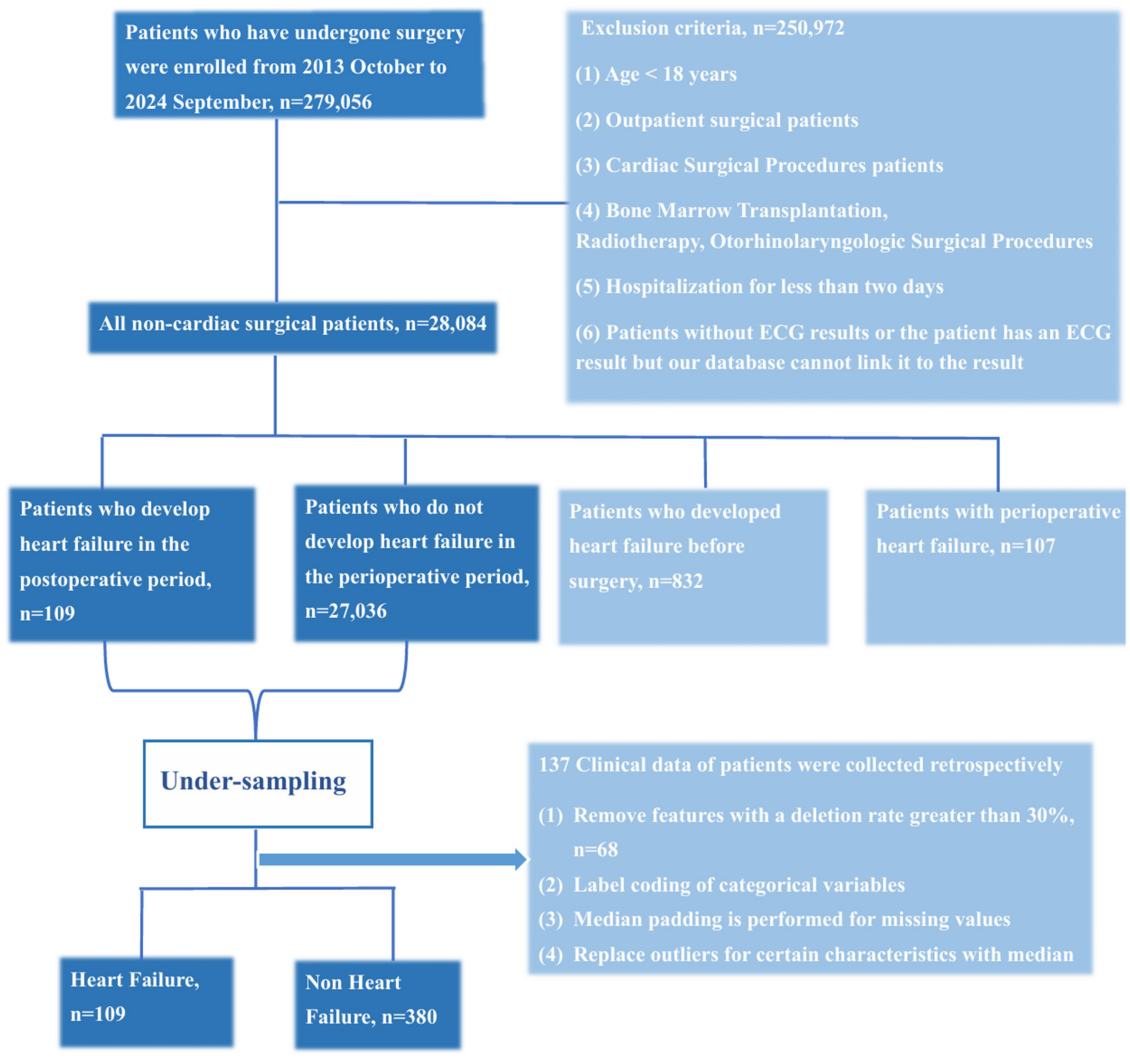
Flow chart of the study population enrollment.

This retrospective cohort study included 109 inpatients who developed heart failure following non-cardiac surgery and 380 inpatients who remained free of heart failure during the perioperative period. All participants were admitted to the Sixth Medical Center of the Chinese PLA General Hospital between October 8, 2013, and September 18, 2024, and underwent at least one non-cardiac surgical procedure during hospitalization. New-onset postoperative heart failure is defined as: heart failure that occurs for the first time after surgery during the index hospitalization in patients without pre-existing chronic heart failure prior to the operation. For external validation, an independent cohort of 10,492 patients—with no overlap with the primary dataset—was obtained from the First Medical Center of the Chinese PLA General Hospital. Inclusion criteria were: (1) age ≥18 years; (2) hospital stay exceeding 2 days with at least one documented non-cardiac surgery; (3) no prior history of cardiac surgery, including valve replacement, coronary artery bypass grafting, or percutaneous coronary intervention; (4) for patients having multiple non-cardiac surgeries, only the first procedure was included. Using SQL, we applied exclusion criteria based on ICD-10 codes (I50.105 for chronic left ventricular dysfunction and I50.908 for chronic heart failure) from preoperative, admission, and historical diagnoses. The final positive cohort consisted solely of patients whom clinicians had assigned a general I50.x diagnosis for heart failure. The patient selection process is illustrated in Figure 1.

### 2.2 Data collection

Clinical variables were retrospectively extracted from the Oracle database of the Sixth Medical Center of the Chinese PLA General Hospital using Structured Query Language (SQL). The collected variables included: (1) *Demographics*: age, sex, and ethnicity; (2) *Comorbidities*: atrial fibrillation, acute coronary syndrome, malignant arrhythmias, primary cardiomyopathies, and others; (3) *Laboratory tests*: hemoglobin, white blood cell count, platelets count, blood urea nitrogen, serum creatinine, glucose, among others; (4) *Vital signs*: heart rate, respiratory rate, systolic blood pressure, and diastolic blood pressure. A total of 137 clinical features were initially extracted. The diagnostic criteria for postoperative new-onset heart failure were based on the ICD-10 codes specified in the《2022 ESC Guidelines on cardiovascular assessment and management of patients undergoing non-cardiac surgery》. These include: I50.0 Congestive heart failure (including I50.000 Congestive heart failure, I50.001 Right heart failure, I50.002 Combined heart failure); I50.1 Left ventricular failure (including I50.100 Left ventricular failure, I50.101 Acute left ventricular failure, I50.102 Left atrial failure, I50.103 Left heart failure with pulmonary edema, I50.104 Cardiac asthma); I50.9 Heart failure, unspecified (including I50.900 Heart failure, I50.906 Myocardial impairment, I50.907 Acute heart failure); and I51.7 Cardiomegaly (including I51.700 × 009 Cardiac enlargement) (10, 11). As a retrospective study, the requirement for informed consent was waived.

### 2.3 Data cleaning and preprocessing

The raw dataset underwent the following preprocessing steps: (1) surgical risk stratification was conducted according to the *2022 ESC Guidelines on Cardiovascular Assessment and Management of Patients Undergoing Non-Cardiac Surgery*. All surgical and interventional procedures performed during hospitalization were categorized into three risk levels (11); (2) features with more than 30% missing values were excluded, reducing total number of features from 137 to 69; (3) categorical variables were converted into numerical format using label encoding; (4) missing values were filled using median imputation via the *Simple Imputer module in Python’s scikit-learn* library. (5) Outliers were identified based on interquartile range (IQR) method [Q1–1.5 × IQR, Q3 + 1.5 × IQR] [Q1–1.5 × IQR, Q3 + 1.5 × IQR] [Q1–1.5 × IQR, Q3 + 1.5 × IQR] and clinical judgment, and were replaced with the median value of the respective feature.

### 2.4 Model development and selection

The dataset was randomly divided into a training set (80%) and a testing set (20%). The training set included 391 patients (87 postoperative heart failure cases, 304 controls), and the testing set comprised 98 patients (22 postoperative heart failure cases, 76 controls). To address class imbalance in the training set, a hybrid method combining random under sampling and Synthetic Minority Oversampling Technique (SMOTE) was used: the negative-to-positive ratio was reduced to 70% by under sampling, followed by Synthetic Minority Oversampling Technique to upsample the minority class. Eight binary classification algorithms were developed: Naive Bayes (NB), K-Nearest Neighbors (KNN), Support Vector Machine (SVM), Logistic Regression (LR), Decision Tree (DT), AdaBoost, XG Boost, and Random Forest (RF). Five-fold cross-validation was employed to enhance model robustness and reduce overfitting. Model performance was evaluated using accuracy, sensitivity, specificity, precision, F1 score (harmonic mean of precision and recall), and area under the receiver operating characteristic curve. The model with the best overall performance was selected for further analysis. Due to the imbalanced distribution of negative and positive samples, the negative samples in the original model were undersampled, and the model was retrained. The model’s calibration was assessed by calculating the Brier score and plotting calibration curves on both internal and external validation datasets. Additionally, we conducted Decision Curve Analysis (DCA) to demonstrate its net clinical benefit and plotted a Clinical Impact Curve (CIC) to determine the optimal threshold probability. This comprehensive visual analysis enabled a rigorous evaluation of the model’s clinical utility and the identification of the most effective decision threshold for practical application.

### 2.5 Feature selection

To improve model interpretability and reduce computational complexity, the most predictive features were identified. Initially, 43 features with an importance score greater than 0.001 were selected based on model-derived feature importance rankings. Next, Recursive Feature Elimination (RFE) with five-fold cross-validation was applied to determine the optimal subset of features. Finally, Pearson correlation analysis was performed to eliminate redundant variables; among highly correlated pairs (correlation coefficient *r* > 0.6), only the most informative feature was retained.

### 2.6 Internal and external validation

To evaluate the generalizability, both internal and external validation were conducted. Internal validation was performed using the held-out testing set comprising 98 patients (22 postoperative heart failure cases and 76 controls). External validation utilized an independent dataset of 5,585 patients from the First Medical Center of the Chinese PLA General Hospital, including 1.536 postoperative heart failure cases and 4,049 controls. This external cohort was used to assess the model’s robustness and applicability across different clinical settings and patient populations. Performance metrics from both validation sets were compared to verify the model’s reliability and clinical utility.

### 2.7 Model interpretability

To enhance model transparency and clinical trust, SHAP were used to interpret prediction results. SHAP values quantify each feature’s contribution to the model output at both global and individual levels, allowing clinicians to understand how specific variables influence risk estimates. This interpretability supports personalized clinical decision-making and facilitates integration of the model into routine practice.

### 2.8 Statistical analysis

All analyses were conducted using Python 3.8.3. Continuous variables were presented as means ± standard deviations, and categorical variables as frequencies and percentages. Between-group comparisons (postoperative heart failure vs. non-postoperative heart failure) were performed using the student’s *t*-test for continuous variables and the chi-square test for categorical variables. Pearson correlation was used to assess associations between continuous variables. A two-sided *p*-value < 0.05 was considered statistically significant.

## 3 Results

### 3.1 Comparison of clinical characteristics

Compared to the non-heart failure group, patients in the HF group had a significantly higher proportion of males (60.6% vs. 48.2%, *p* < 0.05), an older mean age (73.9 vs. 53.8 years, *p* < 0.001), and a greater rate of high-risk surgeries (27.5% vs. 16.3%, *p* < 0.05). Additionally, the HF group had a higher prevalence of comorbidities, including acute coronary syndrome, acute kidney injury, acute exacerbation of chronic obstructive pulmonary disease (AECOPD), atrial fibrillation, chronic kidney disease, coronary artery disease, diabetes or ketoacidosis, myocardial infarction, cor pulmonale, and severe anemia (all *p* < 0.05).

Significant physiological and laboratory differences were also observed: the HF group had lower diastolic blood pressure (70.3 vs. 74.8 mmHg, *p* < 0.001) but higher pulse (89.1 vs. 75.3 bpm, *p* < 0.001) and respiratory rates (21.1 vs. 19.1 breaths/min, *p* < 0.001). Furthermore, they exhibited significantly lower levels of absolute lymphocyte count, albumin, serum calcium, cholesterol, estimated glomerular filtration rate, high-density lipoprotein cholesterol, mean corpuscular hemoglobin concentration, platelet count, serum phosphorus, and total protein, alongside elevated levels of absolute neutrophil count, alkaline phosphatase, activated partial thromboplastin time, blood glucose, direct bilirubin, fibrinogen, gamma-glutamyl transferase, international normalized ratio, neutrophil percentage, neutrophil-to-lymphocyte ratio, prothrombin time, serum creatinine, total bilirubin, and white blood cell count (all *p* < 0.05), as summarized in Table 1.

**Table 1:** Comparison of preoperative baseline characteristics between the two groups of patients.

Characteristics	Overall, *n* = 489	Non-heart failure group, *n* = 380	Heart failure group, *n* = 109	*p*-value
Demographic				
Sex (Female), *n* (%)	240 (49.1)	197 (51.8)	43 (39.4)	0.03
Sex (Male), *n* (%)	249 (50.9)	183 (48.2)	66 (60.6)	
Age, mean (SD)	58.3 (18.6)	53.8 (16.7)	73.9 (16.2)	<0.001
Surgical risk estimate, *n* (%)				
Surgical low risk classification	187 (38.2)	141 (37.1)	46 (42.2)	
Surgical middle risk classification	210 (42.9)	177 (46.6)	33 (30.3)	
Surgical high-risk classification	92 (18.8)	62 (16.3)	30 (27.5)	0.003
Preoperative comorbidities, *n* (%)				
Acute coronary syndrome	7 (1.4)	3 (0.8)	4 (3.7)	0.047
Acute kidney injury	11 (2.2)	3 (0.8)	8 (7.3)	<0.001
Acute poisoning	5 (1.0)	2 (0.5)	3 (2.8)	0.076
AECOPD	4 (0.8)	1 (0.3)	3 (2.8)	0.036
Aortic dissection	3 (0.6)	2 (0.5)	1 (0.9)	0.532
Atrial fibrillation	21 (4.3)	7 (1.8)	14 (12.8)	<0.001
Cardiac arrest	1 (0.2)		1 (0.9)	0.223
Chronic kidney disease	30 (6.1)	8 (2.1)	22 (20.2)	<0.001
Coronary heart disease	86 (17.6)	42 (11.1)	44 (40.4)	<0.001
Diabetes or diabetic ketoacidosis	71 (14.5)	36 (9.5)	35 (32.1)	<0.001
Hypothyroidism	2 (0.4)	1 (0.3)	1 (0.9)	0.396
Infective endocarditis	1 (0.2)		1 (0.9)	0.223
Myocardial infarction	10 (2.0)		10 (9.2)	<0.001
Cor pulmonale	5 (1.0)		5 (4.6)	0.001
Severe anemia	54 (11.0)	24 (6.3)	30 (27.5)	<0.001
Physical, mean (SD)				
BMI	24.1 (3.5)	24.2 (3.6)	23.8 (3.1)	0.26
Pulse	78.4 (13.2)	75.3 (7.0)	89.1 (21.6)	<0.001
Respiratory rate	19.6 (4.0)	19.1 (3.5)	21.1 (5.1)	<0.001
Systolic blood pressure	126.3 (19.0)	125.5 (17.5)	129.1 (23.6)	0.143
Diastolic blood pressure	73.8 (11.3)	74.8 (10.5)	70.3 (13.4)	0.001
Laboratory results, mean (SD)				
Absolute lymphocyte count (*10^9^/L)	1.6 (0.7)	1.8 (0.7)	1.2 (0.6)	<0.001
Absolute monocyte count (*10^9^/L)	0.6 (2.6)	0.5 (0.2)	1.1 (5.6)	0.247
Absolute neutrophil count (*10^9^/L)	5.1 (5.0)	4.0 (2.5)	8.9 (8.6)	<0.001
Alanine aminotransferase (U/L)	38.6 (157.8)	24.5 (32.7)	87.5 (325.1)	0.046
Albumin (g/L)	38.1 (5.8)	39.8 (4.7)	32.1 (5.1)	<0.001
Alkaline phosphatase (U/L)	96.5 (106.2)	88.9 (56.8)	123.1 (196.9)	0.076
APTT(s)	30.8 (6.4)	30.2 (3.3)	32.9 (11.9)	0.023
Blood glucose (mmol/L)	6.3 (2.5)	5.8 (2.0)	8.0 (3.2)	<0.001
Calcium (mmol/L)	2.2 (0.2)	2.2 (0.2)	2.1 (0.2)	<0.001
Chlorine (mmol/L)	105.2 (4.8)	105.2 (4.0)	105.1 (6.9)	0.808
Cholesterol (mmol/L)	4.4 (1.0)	4.6 (0.9)	3.9 (1.2)	<0.001
Direct bilirubin (μmol/L)	8.2 (31.2)	5.5 (21.5)	18.0 (51.5)	0.015
eGFR (mL/min/1.73 m^2^)	104.8 (37.9)	111.4 (31.5)	81.8 (48.2)	<0.001
Fibrinogen quantification (g/L)	3.4 (1.0)	3.3 (0.9)	3.7 (1.3)	0.004
Gamma-glutamyl transferase (U/L)	58.5 (126.8)	49.6 (113.4)	89.5 (162.3)	0.017
HDL-C (mmol/L)	1.2 (0.3)	1.3 (0.3)	1.0 (0.4)	<0.001
Indirect bilirubin (μmol/L)	295.0 (113.5)	294.1 (97.7)	298.2 (157.2)	0.795
INR	1.1 (0.3)	1.0 (0.1)	1.3 (0.5)	<0.001
MCHC (g/L)	331.8 (14.7)	334.1 (13.0)	324.1 (17.4)	<0.001
Neutrophil percentage (%)	63.8 (14.6)	60.0 (12.4)	76.8 (14.4)	<0.001
NLR	4.5 (5.9)	2.9 (3.0)	10.1 (9.1)	<0.001
Platelet count (*10^9^/L)	221.2 (84.9)	226.9 (70.1)	201.2 (121.9)	0.038
Potassium (mmol/L)	4.0 (0.4)	4.0 (0.4)	4.1 (0.6)	0.15
Prothrombin time(s)	11.8 (2.9)	11.2 (1.1)	13.9 (5.4)	<0.001
Serum creatinine (μmol/L)	99.9 (108.0)	82.9 (67.9)	159.0 (178.8)	<0.001
Serum phosphorus (mmol/L)	1.2 (0.3)	1.2 (0.2)	1.1 (0.4)	0.035
Sodium (mmol/L)	139.5 (4.3)	139.5 (3.6)	139.4 (6.3)	0.878
Total bilirubin (μmol/L)	16.5 (37.1)	13.4 (25.4)	27.4 (61.7)	0.022
Total protein (g/L)	65.3 (7.3)	67.2 (6.1)	58.8 (7.6)	<0.001
White blood cell count (*10^9^/L)	7.5 (6.8)	6.4 (2.6)	11.3 (13.0)	<0.001

All comorbidities were pre-existing conditions documented at preoperative admission. Categorical variables are presented as *n* (%). Continuous variables are presented as mean (standard deviation). *p* values less than 0.05 are considered statistically significant. AECOPD, Acute exacerbation of chronic obstructive pulmonary disease; MCHC, mean corpuscular hemoglobin concentration; APTT, activated partial thromboplastin time; INR, International normalized ratio; HDL-C, High-density lipoprotein cholesterol; eGFR, estimated glomerular filtration rate; BMI, body mass index; NLR, neutrophil-to-lymphocyte ratio.

### 3.2 Model construction and performance comparison

**Figure 2:**
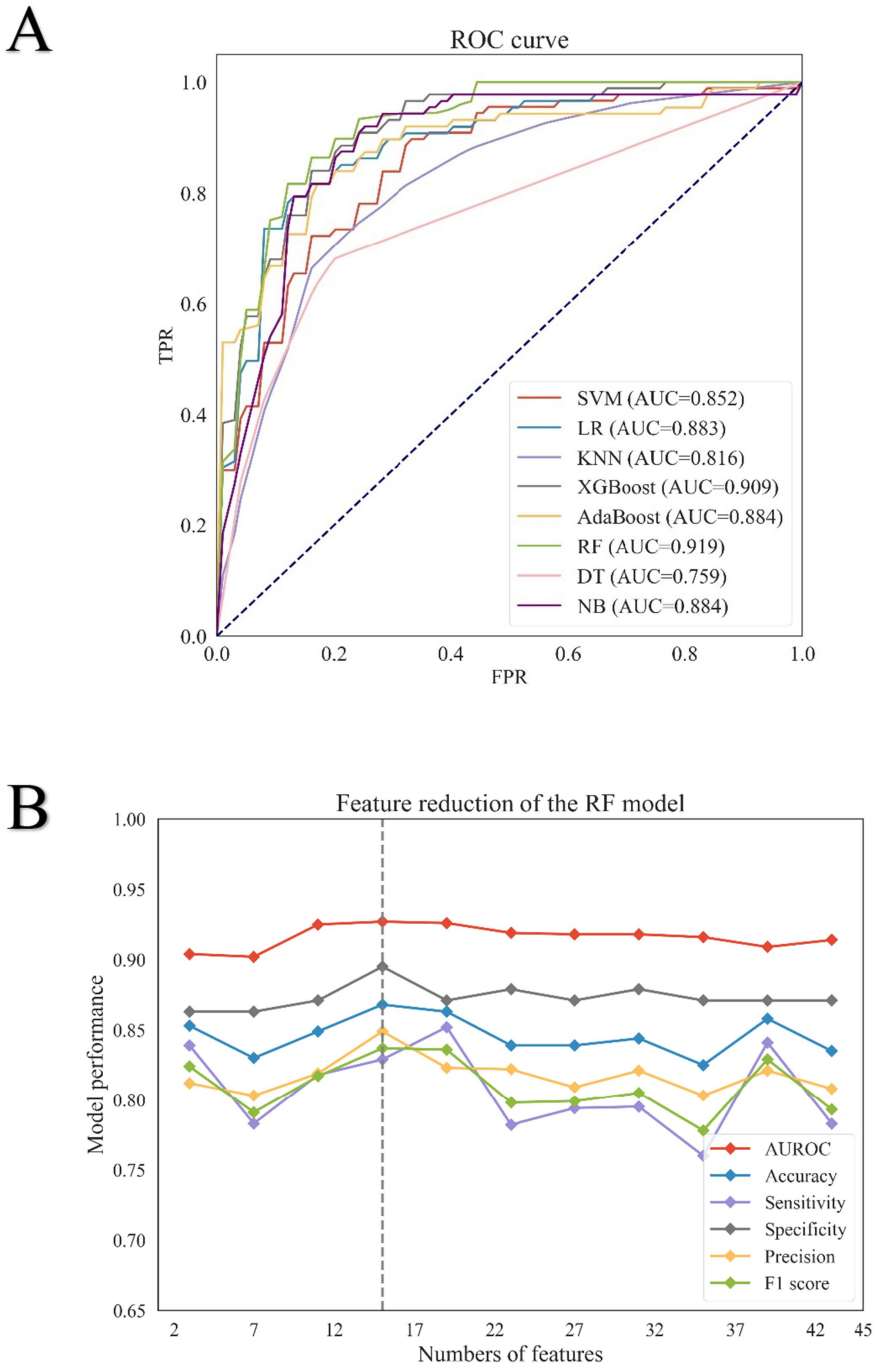
Results of eight machine learning algorithms for screening feature variables. **(A)** Receiver operating characteristic (ROC) curves of eight machine learning models in the training set. The ROC curves illustrate the performance of eight binary classification algorithms: Support Vector Machine (SVM), Logistic Regression (LR), K-Nearest Neighbors (KNN), Extreme Gradient Boosting (XG Boost), AdaBoost, Random Forest (RF), Decision Tree (DT), and Naive Bayes (NB). The ROC curve for SVM is represented by the red line, LR by the blue line, KNN by the light purple line, XG Boost by the dark green line, AdaBoost by the yellow line, RF by the green line, DT by the pink line, and NB by the purple line. **(B)** Feature reduction analysis of the Random Forest (RF) model. Performance metrics of the RF model with varying numbers of features are shown. The area under the receiver operating characteristic curve (AUROC) is represented by a solid red line; accuracy by a solid blue line; sensitivity by a solid purple line; specificity by a solid gray line; precision by a solid yellow line; and F1 score by a solid green line.

Eight binary classification models were developed using the training cohort, based on the following machine learning algorithms: Naive Bayes, K-Nearest Neighbors, Support Vector Machine, Logistic Regression, Decision Tree, AdaBoost, XG Boost, and random forest. Model performance was assessed using five-fold cross-validation, and the average metrics across folds are summarized in Table 2. The corresponding ROC curves are presented in Figure 2A. Among all models, the random forest classifier demonstrated the best overall performance, achieving the highest area under the ROC curve of 0.919, along with an accuracy of 0.849, sensitivity of 0.806, specificity of 0.879, and precision of 0.824. Based on these results, the random forest model was selected as the final predictive model for further validation and interpretation.

**Table 2:** Comparison results of multiple models.

	Accuracy	Sensitivity	Specificity	Precision	F1 score	AUROC	AUPRC
NB	0.806	0.668	0.903	0.829	0.737	0.884	0.847
KNN	0.768	0.635	0.862	0.759	0.683	0.816	0.742
SVM	0.754	0.552	0.896	0.78	0.641	0.852	0.792
LR	0.839	0.771	0.887	0.829	0.797	0.883	0.826
DT	0.778	0.656	0.863	0.779	0.706	0.759	0.788
AdaBoost	0.811	0.76	0.847	0.78	0.768	0.884	0.875
XG Boost	0.815	0.793	0.83	0.773	0.778	0.909	0.873
RF	0.849	0.806	0.879	0.824	0.813	0.919	0.868

NB, Naive Bayes; KNN, K-Nearest Neighbor; SVM, Support Vector Machines; LR, Logistic Regression; DT, Decision Tree; XG-boost, Extreme Gradient Boosting; RF, Random Forest.

### 3.3 Feature variable screening results

Initially, 137 features were collected. After excluding those with over 30% missing data, 69 features remained. Based on importance scores from the random forest model, 43 features with a value greater than 0.001 were retained (Supplementary Figure S1). Recursive feature elimination with five-fold cross-validation was then used to evaluate model performance across different feature subsets (Figure 2B; Supplementary Table S1). Performance metrics—including AUROC, accuracy, specificity, and precision—plateaued after the top 10 features, while sensitivity and F1 score declined. To this end, a comprehensive assessment of the model’s performance at various decision thresholds is provided in Supplementary Table S2. As shown in Supplementary Figure S2, strong correlations were observed between albumin and total protein, among absolute lymphocyte count, absolute neutrophil count, neutrophil percentage, and NLR, and between international normalized ratio (INR) and prothrombin time. As the Neutrophil-to-Lymphocyte Ratio (NLR) is calculated from the absolute neutrophil count and absolute lymphocyte count, these two individual components were removed from the model to avoid multicollinearity. Consequently, albumin, NLR, eGFR, and INR were retained for the final model. Ultimately, 10 features were selected for the final model: age, albumin, neutrophil-to-lymphocyte ratio (NLR), blood glucose, international normalized ratio (INR), pulse rate, mean corpuscular hemoglobin concentration (MCHC), serum creatinine, estimated glomerular filtration rate (eGFR), and diastolic blood pressure. The model’s generalizability was assessed using an external validation cohort, with its calibration performance across various risk deciles showcased in Supplementary Table S3. Furthermore, the Precision-Recall curves for both the internal and external validation cohorts are provided in Supplementary Figures S3 and S4, respectively. To elucidate the interactions between the final predictive features and their collective impact on the model’s output, we performed a SHAP analysis, with the resulting interaction value matrix displayed in Supplementary Figure S5.

### 3.4 Model performance in the validation cohorts

**Figure 3:**
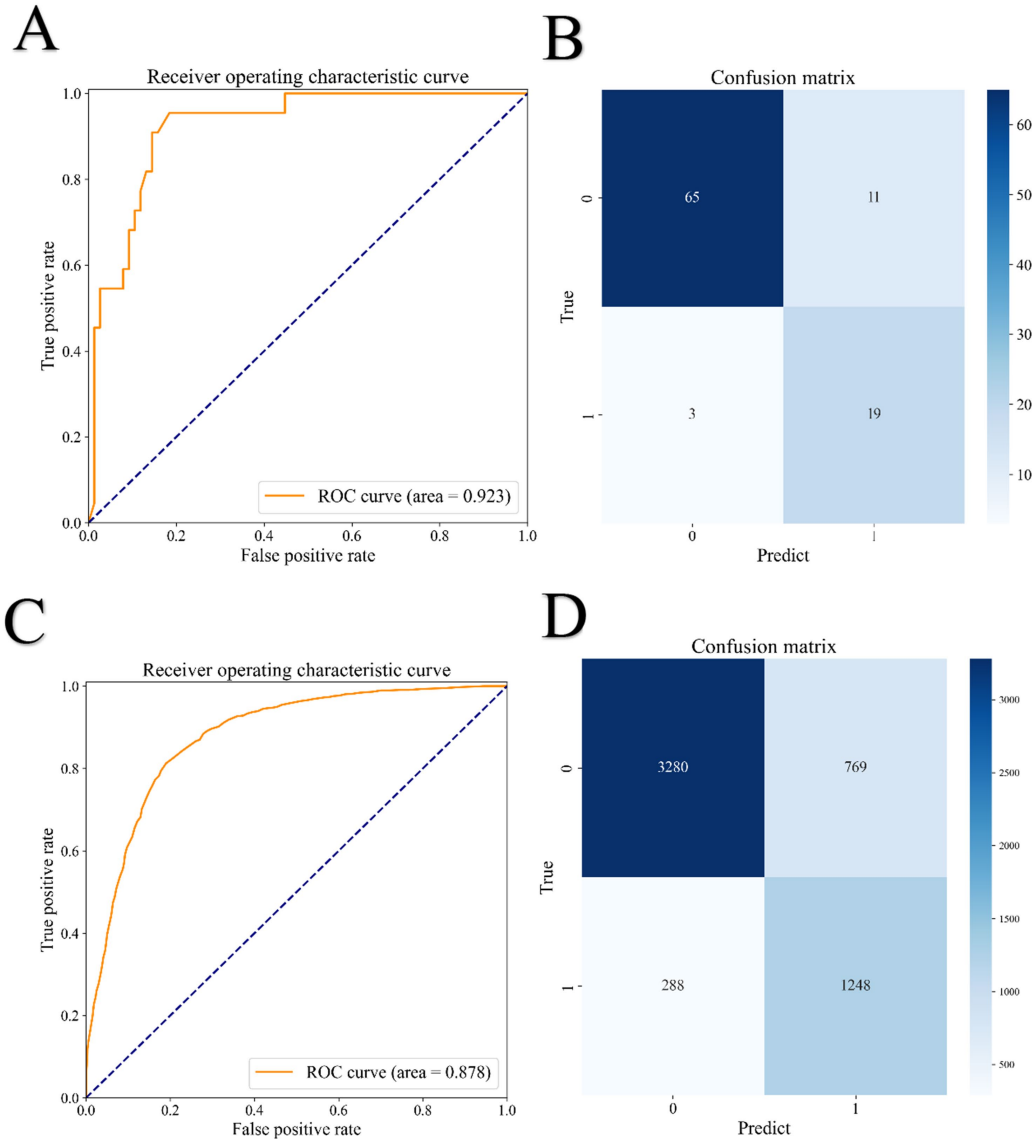
ROC curve and Confusion matrix. **(A)** ROC curve in the internal validation set; **(B)** Confusion matrix in the internal validation set; **(C)** ROC curve in the external validation set; **(D)** Confusion matrix in the external validation set.

The internal validation set comprised 98 samples, comprising 22 positive and 76 negative cases. The final random forest model demonstrated strong performance in this cohort (Figures 3A,B), achieving an accuracy of 0.857, sensitivity of 0.864, specificity of 0.855, precision of 0.633, F1 score of 0.731, and an AUROC of 0.923. Of the 76 negative cases, 65 were correctly classified, while 22 of the 19 positive cases were correctly identified. In the external validation cohort, consisting of 5,585 samples (1,536 positive and 4,049 negative cases), the model achieved an accuracy of 0.619, sensitivity of 0.813, specificity of 0.810, and an AUROC of 0.878 (Figure 3C). A total of 3,280 out of 4,049 negative cases and 1,248 out of 1,536 positive cases were correctly predicted (Figure 3D).

### 3.5 Clinical utility assessment of the predictive model

To comprehensively evaluate the clinical value of our predictive model, we performed calibration, Decision Curve Analysis (DCA), and Clinical Impact Curve (CIC) analysis on the Random Forest (RF) model.

**Figure 4:**
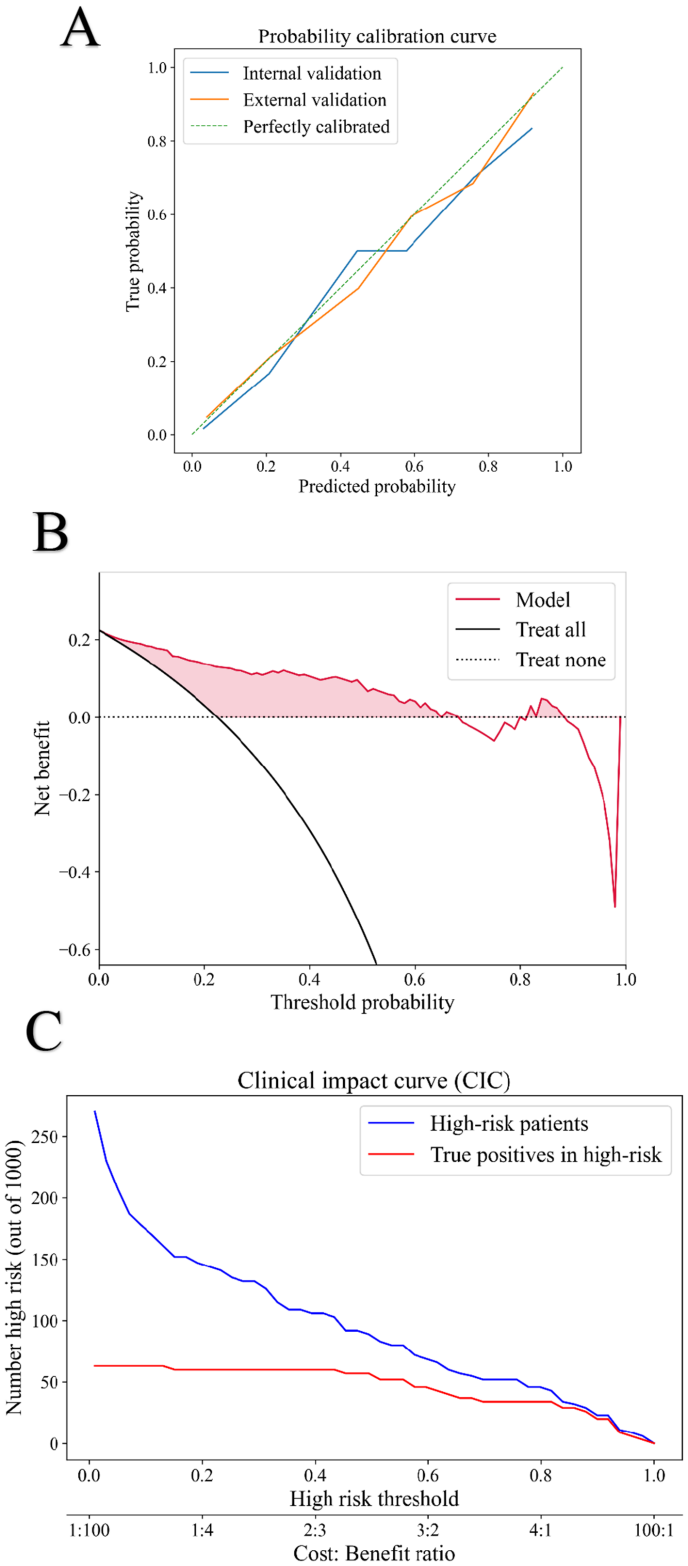
Calibration capability and clinical benefit of the model. **(A)** The calibration curve in the internal and external validation of the RF model. The blue solid line represents the internal validation set, and the orange solid line represents the external validation set. **(B)** DCA of the RF model. **(C)** CIC of the RF model; Among a cohort of 1,000 patients, the blue solid line represents the number of individuals classified as high risk by the model at each risk threshold. The red solid line indicates the number of true positive cases within that group.

As shown in Figure 4A, the calibration curves for both the internal test set and external validation set (the x-axis represents the predicted risk of postoperative heart failure, while the y-axis indicates the observed actual incidence) demonstrated good agreement between predictions and observations, with the RF model’s performance (solid line) closely following the ideal diagonal (dashed line). This was supported by Brier scores of 0.093 and 0.123 for internal and external validation, respectively. The DCA (Figure 4B) revealed that across a threshold probability range of 0–1.0, the RF model provided a superior net clinical benefit compared to the strategies of “intervening on all” or “none.” The CIC (Figure 4C) further illustrated the model’s performance across thresholds. In a sample of 1,000 patients, the number of patients identified as high-risk by the model (blue curve) closely approximated the actual number of true positive cases (red curve) as the risk threshold increased.

Based on this comprehensive analysis, a threshold probability of 0.6 was selected as optimal for clinical decision-making. This threshold effectively balances sensitivity and specificity, minimizing both unnecessary interventions from false positives and the risk of missing true positive cases.

### 3.6 Interpretability analysis of the model

**Figure 5:**
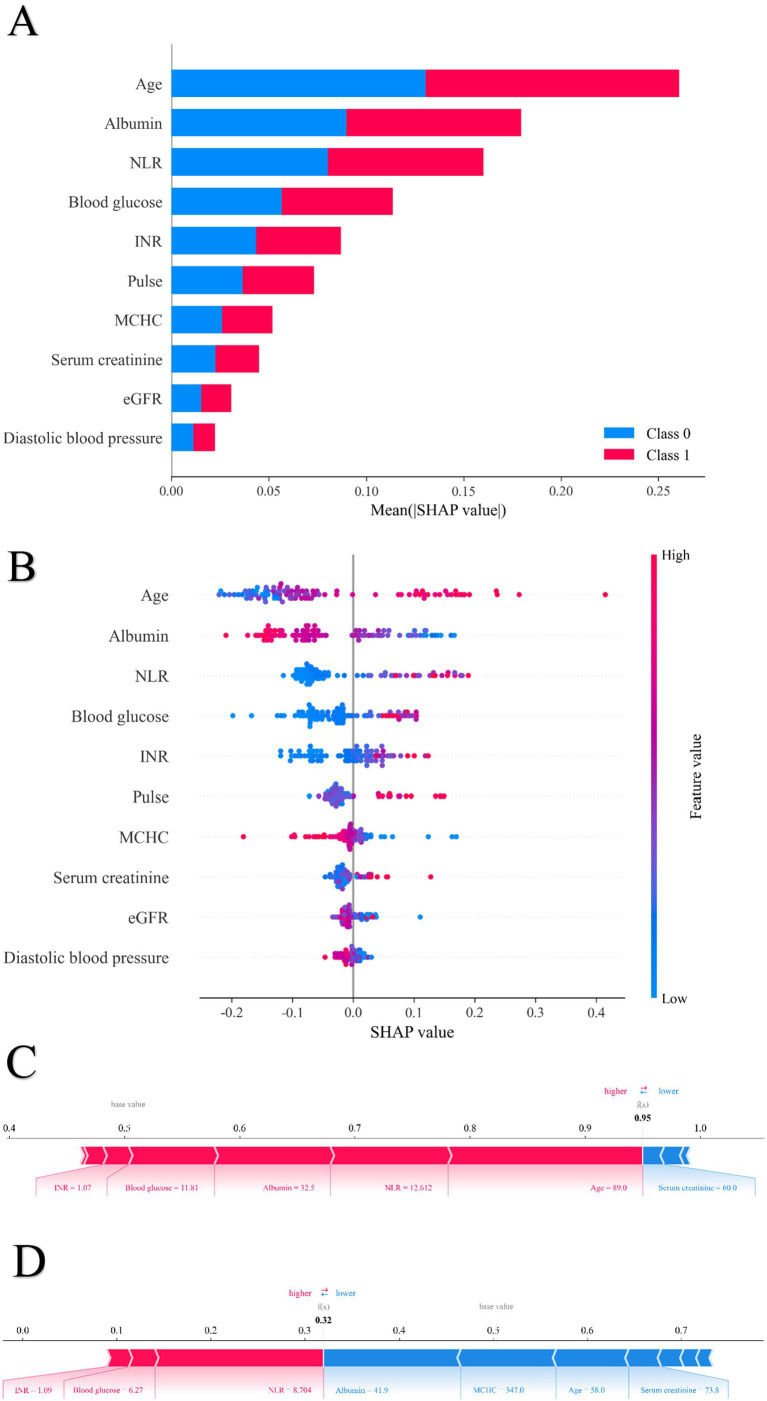
The SHAP value of each variable for sample in the random forest model. **(A)** Order plot of variable importance for SHAP analysis; **(B)** Statistical graph of variable contribution in SHAP analysis; **(C)** Individual efforts by patients with heart failure; **(D)** Individual efforts by patients without heart failure.

**Figure 6:**
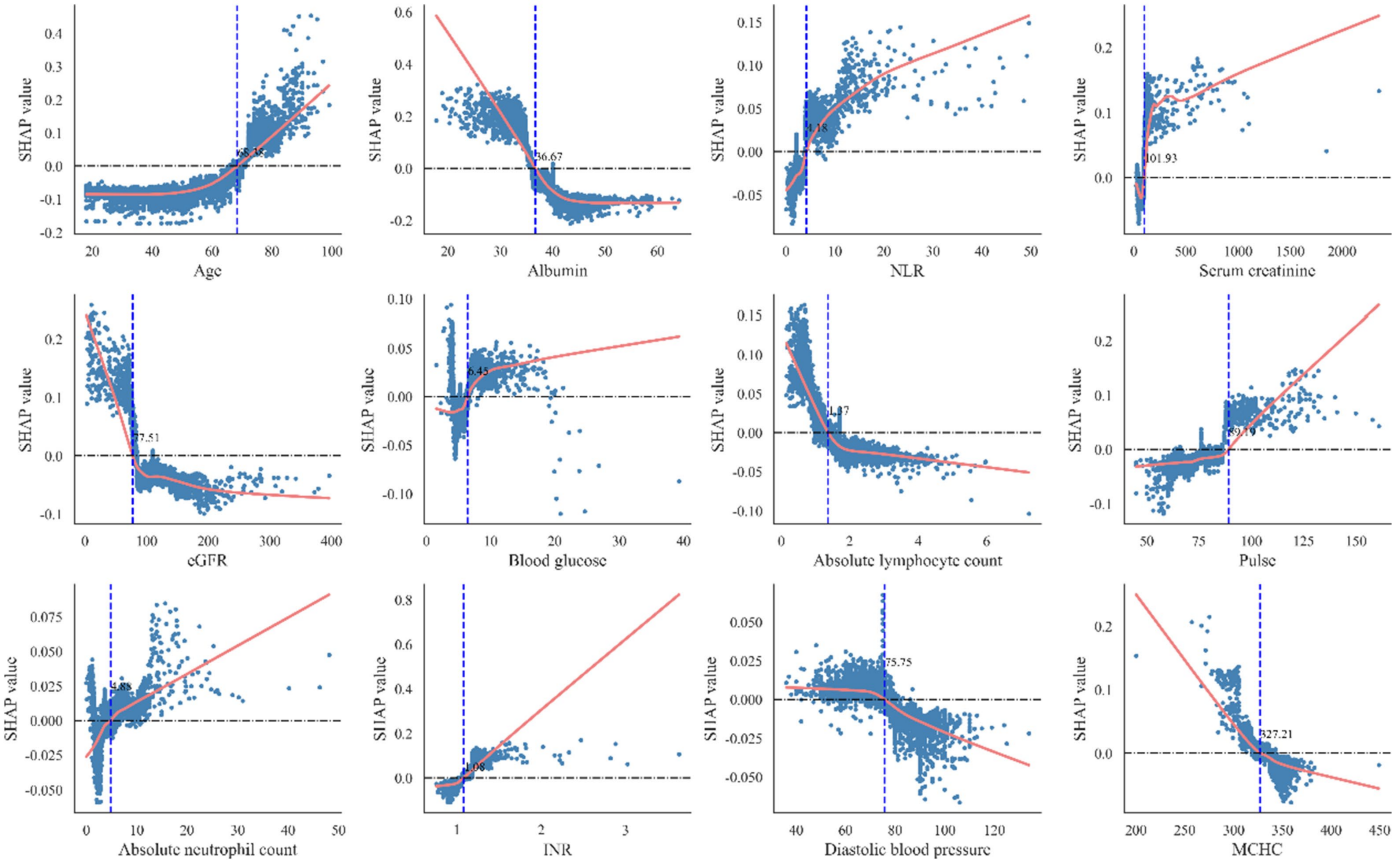
The optimal classification model obtains the best cutoff point of risk factors for included patients. INR, International normalized ratio; eGFR, estimated glomerular filtration rate; NLR, neutrophil-to-lymphocyte ratio. MCHC, Mean Corpuscular Hemoglobin Concentration.

To enhance interpretability and identify key factors influencing postoperative heart failure following non-cardiac surgery, SHAP was applied to analyze the random forest model. Feature importance rankings based on SHAP values are shown in Figure 5A, highlighting albumin, age, and estimated glomerular filtration rate as the most influential predictors among the 10 selected features. The mean absolute SHAP values for the 10 predictors are as follows: Age: 0.27, Albumin: 0.18, NLR: 0.16, Blood Glucose: 0.12, INR: 0.08, Pulse Rate: 0.07, MCHC: 0.05, Serum Creatinine: 0.04, eGFR: 0.03, Diastolic Blood Pressure: 0.02. Figure 5B presents the distribution of SHAP values for each feature. The x-axis represents the SHAP value, and dot color indicates the magnitude of the corresponding feature value (red = high, blue = low). A positive SHAP value indicates increased predicted risk of postoperative heart failure, while a negative value suggests a protective effect. The model’s predictions were primarily driven by age, which showed a strong positive correlation with the risk of pHF. This positive association was also shared by NLR, blood glucose, INR, pulse rate, and serum creatinine. In contrast, albumin, MCHC, eGFR, and diastolic blood pressure had a negative correlation, implying a protective effect, as lower values of these features corresponded to a higher pHF risk. Beyond global feature influence, SHAP analysis also enabled interpretation at the individual prediction level. Feature contributions for specific cases are illustrated in Figure 5C (positive case) and Figure 5D (negative case). In the positive case, features such as mean corpuscular hemoglobin concentration, blood glucose, and neutrophil-to-lymphocyte ratio contributed to increased postoperative heart failure risk. Conversely, in the negative case, albumin, age, and estimated glomerular filtration rate were associated with lower risk. The SHAP dependence plots were further used to visualize the impact of individual features on predictions. In these plots, the x-axis represents feature values, and the y-axis indicates SHAP values, with a LOWESS-smoothed line showing the overall trend. As shown in Figure 6, The Random Forest model identified specific inflection points for key predictive features, beyond which the risk of postoperative heart failure notably changed. These critical values were: Age: 68.92 years; Albumin: 36.59 g/L; NLR: 5.18; Serum Creatinine: 104.96 μmol/L; eGFR: 82.64 mL/min/1.73 m^2^; Blood Glucose: 5.85 mmol/L; Pulse Rate: 86.53 beats/min; INR: 1.04; Diastolic Blood Pressure: 72 mmHg; and MCHC: 330.52 g/L. It is noteworthy that these values represent the physiological ranges at which the model identifies a steep increase in the risk of postoperative heart failure; however, they themselves are not clinically optimized decision thresholds.

### 3.7 Application of model

To enhance clinical utility, the final model (AUROC: 0.923) was optimized and deployed as a user-friendly, web-based application.^1^ This streamlined tool enables clinicians to input recent preoperative laboratory values for rapid estimation of postoperative heart failure risk. In addition, the application provides SHAP-based visual explanations of individual predictions, offering clinicians transparent insights into how each feature contributes to the risk estimate and supporting informed clinical decision-making.

## 4 Discussion

Previous studies have reported an incidence of primary acute heart failure of approximately 2.5% following surgery, while patients with pre-existing chronic heart failure exhibit a postoperative one-year mortality rate as high as 52% (12). In the present study, the incidence of perioperative heart failure after non-cardiac surgery was 3.7%, with an in-hospital mortality rate of 61.5% among those who developed heart failure postoperatively. These findings highlight the urgent need for accurate preoperative risk assessment to identify high-risk individuals and enable timely, targeted interventions aimed at reducing the risk of postoperative heart failure. Existing perioperative cardiac risk stratification tools predominantly focus on predicting myocardial infarction or cardiac arrest in patients with known chronic heart failure, without specifically addressing postoperative heart failure (5, 13–15). Compared to patients with pre-existing chronic heart failure, those who experience postoperative heart failure have received considerably less clinical attention—particularly in the context of non-cardiac surgery, where cardiovascular specialists are frequently not involved in perioperative management. This lack of specialized oversight may contribute to delayed diagnosis, disease progression, and poor outcomes in this vulnerable population.

Recent advances in AI have opened new avenues for the prevention, diagnosis, and management of cardiovascular diseases (16). AI-based tools, such as the AI-ECG risk estimator (AIRE) and predictive models for postoperative atrial fibrillation, exemplify the growing utility of machine learning in this domain (7, 17, 18). Machine learning-based risk prediction models offer substantial advantages by integrating multidimensional preoperative data—such as values and vital signs—into objective, quantifiable predictors. This approach overcomes the limitations of traditional models, which often rely on clinical judgment and subjective symptom reporting. By mining large-scale medical datasets, machine learning algorithms are capable of capturing latent patterns of disease progression and identifying critical risk factors (19, 20), thereby improving predictive accuracy, enhancing model robustness, and enabling generalizability across diverse clinical contexts.

In this study, we developed and compared eight machine learning-based models for predicting heart failure following non-cardiac surgery. Among these, the random forest algorithm—a widely adopted ensemble learning technique—demonstrated superior discriminative ability. Random forest constructs multiple decision trees and aggregates their predictions to improve accuracy, generalizability, and resistance to overfitting. The random forest model in our study exhibited excellent predictive performance, especially in identifying high-risk patients, and maintained strong predictive capability in the external validation cohort (AUC = 0.878). To improve model interpretability, SHAP were employed to visualize the contribution of individual features to the prediction outcomes. The SHAP analysis identified age, albumin, neutrophil-to-lymphocyte ratio (NLR), blood glucose, international normalized ratio (INR), pulse rate, mean corpuscular hemoglobin concentration (MCHC), serum creatinine, estimated glomerular filtration rate (eGFR), and diastolic blood pressure as key predictors of postoperative heart failure.

Age emerged as another critical predictor of postoperative heart failure. Advancing biological age is associated with reductions in left ventricular mass, chamber volume, and cardiac output predispose patients to heart failure (21). In addition, older individuals having non-cardiac surgery face increased hemodynamic stress during anesthesia and surgical procedures, which further raises the risk of postoperative cardiac events (1, 22). These findings support the need for personalized, proactive preoperative assessments and interventions strategies in elderly patients, and underscore the value of age-specific approaches to heart failure risk prediction. Hypoalbuminemia was strongly and negatively associated with the risk of postoperative heart failure. Poor nutritional status—often resulting from cachexia, frailty, or impaired immune function—can lead to preoperative hypoalbuminemia. In cases of significant fluid retention, dilutional hypoalbuminemia may also occur. The relationship between albumin levels and volume status may partly explain its predictive value for heart failure (23–25). Pathophysiology perioperative hypoalbuminemia contributes to pulmonary edema by lowering plasma oncotic pressure (26). Additionally, the coexistence of hypoalbuminemia and water-sodium retention can exacerbate volume overload and reduce responsiveness to diuretics (27).

Elevated preoperative neutrophil-to-lymphocyte ratio were associated with an increased postoperative heart failure risk. Physiological stress and acute illness may activate the sympathetic nervous system and the hypothalamic–pituitary–adrenal (HPA) axis, resulting in elevated cortisol and catecholamine levels that promote neutrophil release. Inflammatory states—commonly induced by trauma or malignancy—can trigger damage-associated molecular patterns (DAMPs), further driving neutrophil recruitment (28). These immune and stress responses, including increased tumor necrosis factor-1 (TNF-1), may also suppress lymphocyte counts and elevate neutrophil-to-lymphocyte ratio (29). Neutrophil-derived enzymes and reactive oxygen species may mediate myocardial remodeling and injury, thus promoting postoperative heart failure onset.

Our findings demonstrated a positive correlation between elevated blood glucose levels and the risk of heart failure (HF). Excessively high blood glucose is implicated in increased disordered cellular calcium metabolism and cardiomyocyte apoptosis, leading to structural changes in the heart that impair myocardial relaxation and cause ventricular stiffening (30–33). This suggests that preoperative blood glucose should be closely monitored and endocrine management optimized to mitigate perioperative HF risk by avoiding hyperglycemia. An elevated international normalized ratio also emerged as a significant predictor of postoperative heart failure. This may reflect not only coagulopathy but also systemic inflammation, venous congestion–related hemodilution, and hepatic dysfunction—factors known to contribute to heart failure pathogenesis (34). A higher resting heart rate was identified as a key risk factor. Tachycardia, often triggered by surgical trauma, hemorrhage, or systemic inflammation, may lead to increased neurohormonal activation, impaired coronary perfusion, reduced contractility, and elevated myocardial oxygen demand (35, 36). Addressing preoperative tachycardia may therefore represent a modifiable target to reduce the risk of postoperative heart failure.

Low mean corpuscular hemoglobin concentration, a marker of anemia, was identified as another relevant predictor of postoperative heart failure. Preoperative anemia—due to malnutrition, chronic bleeding, or trauma—may increase cardiac workload by activating the sympathetic and renin–angiotensin systems, inducing ventricular remodeling and systolic dysfunction (37, 38). Anemia also increases the likelihood of intraoperative transfusions, further contributing to volume overload (39). The coexistence of chronic kidney disease and heart failure is well-established, largely due to shared pathophysiological mechanisms (40). Declining estimated glomerular filtration rate and elevated serum creatinine reflect renal impairment and are associated with poorer outcomes in patients with heart failure (41, 42). In the perioperative setting, fluid losses resulting from surgical procedures are often managed with intravenous fluids or blood transfusions. However, in patients with chronic kidney disease, impaired natriuretic and diuretic capacity may lead to fluid overload, placing additional strain on the heart. Moreover, chronic kidney disease patients are more vulnerable to electrolyte imbalances caused by fasting, hemorrhage, or anesthetic agents, which can impair myocardial contractility and increase the risk of cardiac injury (43). Low diastolic blood pressure was also associated with elevated postoperative heart failure risk. Diastolic blood pressure reduction may result from hemorrhage or anesthetic-induced vasodilation (44, 45). Since coronary perfusion occurs during diastole, low diastolic blood pressure can impair myocardial oxygen delivery and cause ischemic injury (46). Aggressive fluid or transfusion therapy to correct hypotension may worsen cardiac volume overload (47). Thus, individualized anesthesia and fluid management strategies are warranted.

In summary, preoperative identification and optimization of high-risk patients—such as those with malnutrition, advanced age, or renal dysfunction—are essential. The machine learning model developed in this study provides a robust and interpretable tool for perioperative risk stratification and resource allocation.

The model demonstrated strong external validation performance (AUC = 0.878), indicating good generalizability. As the selected predictors are routine clinical parameters—readily available and easily measurable—the model has strong potential for implementation across diverse healthcare settings. Additionally, a user-friendly web-based tool was developed to support individualized postoperative heart failure risk estimation and enhance clinical decision-making and resource allocation.

Despite its strengths, this study has limitations. First, the retrospective design and relatively small number of positive cases may limit generalizability compared to larger cohorts. Second, external validation was conducted at a single center in Beijing, which may constrain geographic applicability. Future multicenter prospective studies are needed to further validate model performance and enhance specificity by incorporating additional clinical variables.

## 5 Conclusion

We developed eight machine learning-based models to predict heart failure following non-cardiac surgery and identified the random forest model as achieving the highest predictive performance. The 10 selected features reflect patients’ preoperative physiological status and offer clinicians practical and interpretable tool to support perioperative decision-making and reduce the incidence of postoperative heart failure.

## Data Availability

The original contributions presented in the study are included in the article/Supplementary material, further inquiries can be directed to the corresponding author.

## Footnotes

1
https://adultv2.streamlit.app/
