# Supplementary material for: Development and validation of an interpretable machine learning model for predicting the risk of non-cardiac surgery postoperative heart failure: a multicenter study
Source: Front Med (Lausanne). 2025 Dec 11;12:1666885. doi: 10.3389/fmed.2025.1666885 (PMC12738863; doi:10.3389/fmed.2025.1666885)
Supplement: Supplementary file 1 [file Data_Sheet_1.docx]

***Supplementary Material***

**Table S1.**

The recursive elimination method is used to evaluate the performance of the model

| 43 | 0.849 | 0.793 | 0.883 | 0.803 | 0.796 | 0.921 | 0.85 |
| --- | --- | --- | --- | --- | --- | --- | --- |
| 42 | 0.853 | 0.816 | 0.876 | 0.806 | 0.807 | 0.929 | 0.886 |
| 41 | 0.836 | 0.78 | 0.869 | 0.781 | 0.779 | 0.926 | 0.88 |
| 40 | 0.844 | 0.803 | 0.869 | 0.786 | 0.794 | 0.922 | 0.871 |
| 39 | 0.866 | 0.827 | 0.89 | 0.822 | 0.822 | 0.932 | 0.893 |
| 38 | 0.862 | 0.849 | 0.869 | 0.802 | 0.823 | 0.928 | 0.885 |
| 37 | 0.853 | 0.816 | 0.876 | 0.8 | 0.806 | 0.927 | 0.883 |
| 36 | 0.845 | 0.804 | 0.869 | 0.785 | 0.794 | 0.921 | 0.865 |
| 35 | 0.84 | 0.803 | 0.862 | 0.779 | 0.79 | 0.917 | 0.866 |
| 34 | 0.858 | 0.827 | 0.876 | 0.803 | 0.813 | 0.926 | 0.873 |
| 33 | 0.866 | 0.839 | 0.883 | 0.816 | 0.824 | 0.932 | 0.892 |
| 32 | 0.866 | 0.826 | 0.89 | 0.823 | 0.823 | 0.926 | 0.884 |
| 31 | 0.857 | 0.815 | 0.883 | 0.808 | 0.811 | 0.935 | 0.902 |
| 30 | 0.853 | 0.839 | 0.862 | 0.79 | 0.81 | 0.925 | 0.88 |
| 29 | 0.849 | 0.816 | 0.869 | 0.792 | 0.801 | 0.926 | 0.881 |
| 28 | 0.849 | 0.816 | 0.869 | 0.79 | 0.801 | 0.923 | 0.873 |
| 27 | 0.849 | 0.815 | 0.869 | 0.789 | 0.801 | 0.932 | 0.892 |
| 26 | 0.862 | 0.827 | 0.883 | 0.809 | 0.818 | 0.936 | 0.902 |
| 25 | 0.858 | 0.85 | 0.862 | 0.789 | 0.818 | 0.933 | 0.897 |
| 24 | 0.862 | 0.85 | 0.869 | 0.799 | 0.822 | 0.925 | 0.874 |
| 23 | 0.858 | 0.838 | 0.869 | 0.798 | 0.816 | 0.924 | 0.868 |
| 22 | 0.853 | 0.839 | 0.862 | 0.787 | 0.811 | 0.927 | 0.88 |
| 21 | 0.853 | 0.826 | 0.869 | 0.8 | 0.809 | 0.925 | 0.878 |
| 20 | 0.87 | 0.861 | 0.876 | 0.811 | 0.834 | 0.932 | 0.879 |
| 19 | 0.849 | 0.827 | 0.862 | 0.787 | 0.804 | 0.924 | 0.879 |
| 18 | 0.879 | 0.861 | 0.89 | 0.825 | 0.842 | 0.927 | 0.876 |
| 17 | 0.853 | 0.826 | 0.869 | 0.794 | 0.809 | 0.926 | 0.882 |
| 16 | 0.858 | 0.827 | 0.876 | 0.807 | 0.814 | 0.925 | 0.883 |
| 15 | 0.87 | 0.873 | 0.869 | 0.805 | 0.836 | 0.931 | 0.888 |
| 14 | 0.853 | 0.839 | 0.862 | 0.79 | 0.813 | 0.927 | 0.881 |
| 13 | 0.866 | 0.85 | 0.876 | 0.809 | 0.828 | 0.925 | 0.885 |
| 12 | 0.853 | 0.838 | 0.862 | 0.788 | 0.811 | 0.927 | 0.88 |
| 11 | 0.858 | 0.85 | 0.862 | 0.79 | 0.818 | 0.93 | 0.884 |
| 10 | 0.858 | 0.839 | 0.869 | 0.802 | 0.816 | 0.914 | 0.864 |
| 9 | 0.836 | 0.816 | 0.848 | 0.77 | 0.79 | 0.915 | 0.862 |
| 8 | 0.836 | 0.816 | 0.848 | 0.769 | 0.79 | 0.91 | 0.859 |
| 7 | 0.823 | 0.792 | 0.841 | 0.756 | 0.771 | 0.909 | 0.849 |
| 6 | 0.828 | 0.816 | 0.834 | 0.752 | 0.781 | 0.903 | 0.845 |
| 5 | 0.845 | 0.839 | 0.848 | 0.779 | 0.804 | 0.901 | 0.828 |
| 4 | 0.823 | 0.817 | 0.828 | 0.746 | 0.773 | 0.889 | 0.828 |
| 3 | 0.798 | 0.748 | 0.828 | 0.733 | 0.735 | 0.87 | 0.797 |
| 2 | 0.785 | 0.782 | 0.786 | 0.693 | 0.731 | 0.88 | 0.822 |
| 1 | 0.638 | 0.633 | 0.641 | 0.512 | 0.562 | 0.69 | 0.612 |

**Table S2**

Comprehensive Performance Metrics at Various Decision Thresholds for the Random Forest Model

| Threshold | TP | FP | TN | FN | PPV | PPV CI low | PPV CI high | PPV 95% CI | NPV | NPV CI low | NPV CI high | NPV 95% CI |
| --- | --- | --- | --- | --- | --- | --- | --- | --- | --- | --- | --- | --- |
| 0.05 | 22 | 54 | 22 | 0 | 0.2895 | 0.1923 | 0.3919 | [0.1923:0.3919] | 1 | 1 | 1 | [1:1] |
| 0.1 | 22 | 40 | 36 | 0 | 0.3548 | 0.2343 | 0.4821 | [0.2343:0.4821] | 1 | 1 | 1 | [1:1] |
| 0.15 | 21 | 34 | 42 | 1 | 0.3818 | 0.2632 | 0.5098 | [0.2632:0.5098] | 0.9767 | 0.9167 | 1 | [0.9167:1] |
| 0.2 | 21 | 30 | 46 | 1 | 0.4118 | 0.2857 | 0.5582 | [0.2857:0.5582] | 0.9787 | 0.9318 | 1 | [0.9318:1] |
| 0.25 | 21 | 27 | 49 | 1 | 0.4375 | 0.2856 | 0.5898 | [0.2856:0.5898] | 0.98 | 0.9375 | 1 | [0.9375:1] |
| 0.3 | 21 | 25 | 51 | 1 | 0.4565 | 0.3191 | 0.5957 | [0.3191:0.5957] | 0.9808 | 0.9344 | 1 | [0.9344:1] |
| 0.35 | 21 | 19 | 57 | 1 | 0.525 | 0.3783 | 0.6775 | [0.3783:0.6775] | 0.9828 | 0.9444 | 1 | [0.9444:1] |
| 0.4 | 21 | 16 | 60 | 1 | 0.5676 | 0.4165 | 0.7317 | [0.4165:0.7317] | 0.9836 | 0.9454 | 1 | [0.9454:1] |
| 0.45 | 21 | 14 | 62 | 1 | 0.6 | 0.4412 | 0.7692 | [0.4412:0.7692] | 0.9841 | 0.9491 | 1 | [0.9491:1] |
| 0.5 | 20 | 11 | 65 | 2 | 0.6452 | 0.4828 | 0.8148 | [0.4828:0.8148] | 0.9701 | 0.9219 | 1 | [0.9219:1] |
| 0.55 | 18 | 10 | 66 | 4 | 0.6429 | 0.4615 | 0.815 | [0.4615:0.815] | 0.9429 | 0.8769 | 0.9867 | [0.8769:0.9867] |
| 0.6 | 16 | 8 | 68 | 6 | 0.6667 | 0.4782 | 0.85 | [0.4782:0.85] | 0.9189 | 0.8485 | 0.9733 | [0.8485:0.9733] |
| 0.65 | 14 | 7 | 69 | 8 | 0.6667 | 0.4736 | 0.8636 | [0.4736:0.8636] | 0.8961 | 0.8209 | 0.9589 | [0.8209:0.9589] |
| 0.7 | 12 | 6 | 70 | 10 | 0.6667 | 0.4286 | 0.8752 | [0.4286:0.8752] | 0.875 | 0.8023 | 0.9481 | [0.8023:0.9481] |
| 0.75 | 12 | 6 | 70 | 10 | 0.6667 | 0.4444 | 0.8667 | [0.4444:0.8667] | 0.875 | 0.7952 | 0.9439 | [0.7952:0.9439] |
| 0.8 | 12 | 4 | 72 | 10 | 0.75 | 0.5263 | 0.9412 | [0.5263:0.9412] | 0.878 | 0.8095 | 0.9405 | [0.8095:0.9405] |
| 0.85 | 10 | 1 | 75 | 12 | 0.9091 | 0.7143 | 1 | [0.7143:1] | 0.8621 | 0.7857 | 0.9302 | [0.7857:0.9302] |
| 0.9 | 7 | 1 | 75 | 15 | 0.875 | 0.5714 | 1 | [0.5714:1] | 0.8333 | 0.7556 | 0.9043 | [0.7556:0.9043] |
| 0.95 | 3 | 1 | 75 | 19 | 0.75 | 0 | 1 | [0:1] | 0.7979 | 0.7097 | 0.8646 | [0.7097:0.8646] |

**Table S3**

Calibration Performance of the Random Forest Model Across Risk Deciles in the External Validation Cohort

| Decile | N-Patients | Mean-Predicted-Risk | Observed Events | Expected Events | OE Ratio |
| --- | --- | --- | --- | --- | --- |
| 1 | 18 | 0.0061 | 0 | 0.1097 | 0 |
| 2 | 12 | 0.0144 | 0 | 0.173 | 0 |
| 4 | 11 | 0.02 | 0 | 0.2198 | 0 |
| 5 | 11 | 0.0513 | 1 | 0.5648 | 1.7704 |
| 6 | 8 | 0.1047 | 0 | 0.838 | 0 |
| 7 | 8 | 0.2499 | 2 | 1.9991 | 1.0005 |
| 8 | 10 | 0.5056 | 6 | 5.056 | 1.1867 |
| 9 | 10 | 0.6859 | 4 | 6.8595 | 0.5831 |
| 10 | 10 | 0.8625 | 9 | 8.6251 | 1.0435 |

**
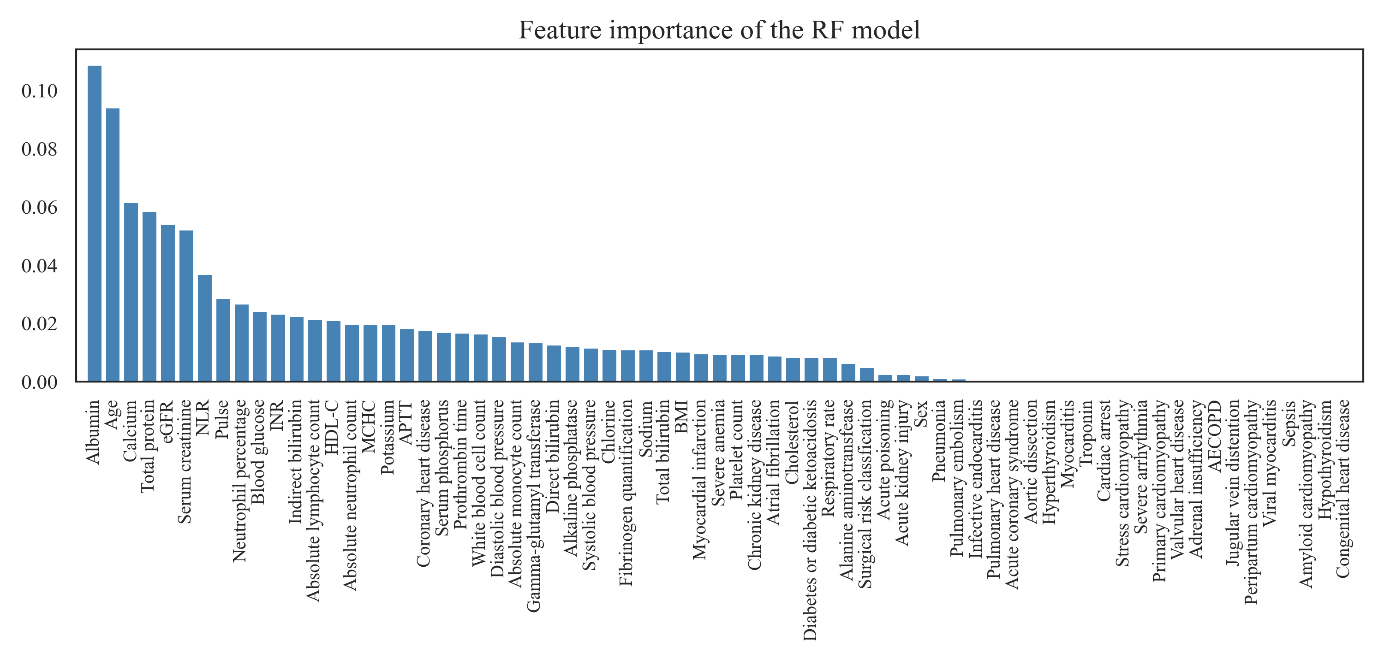
**

**Figure S1.** Feature importance of the RF model.

**
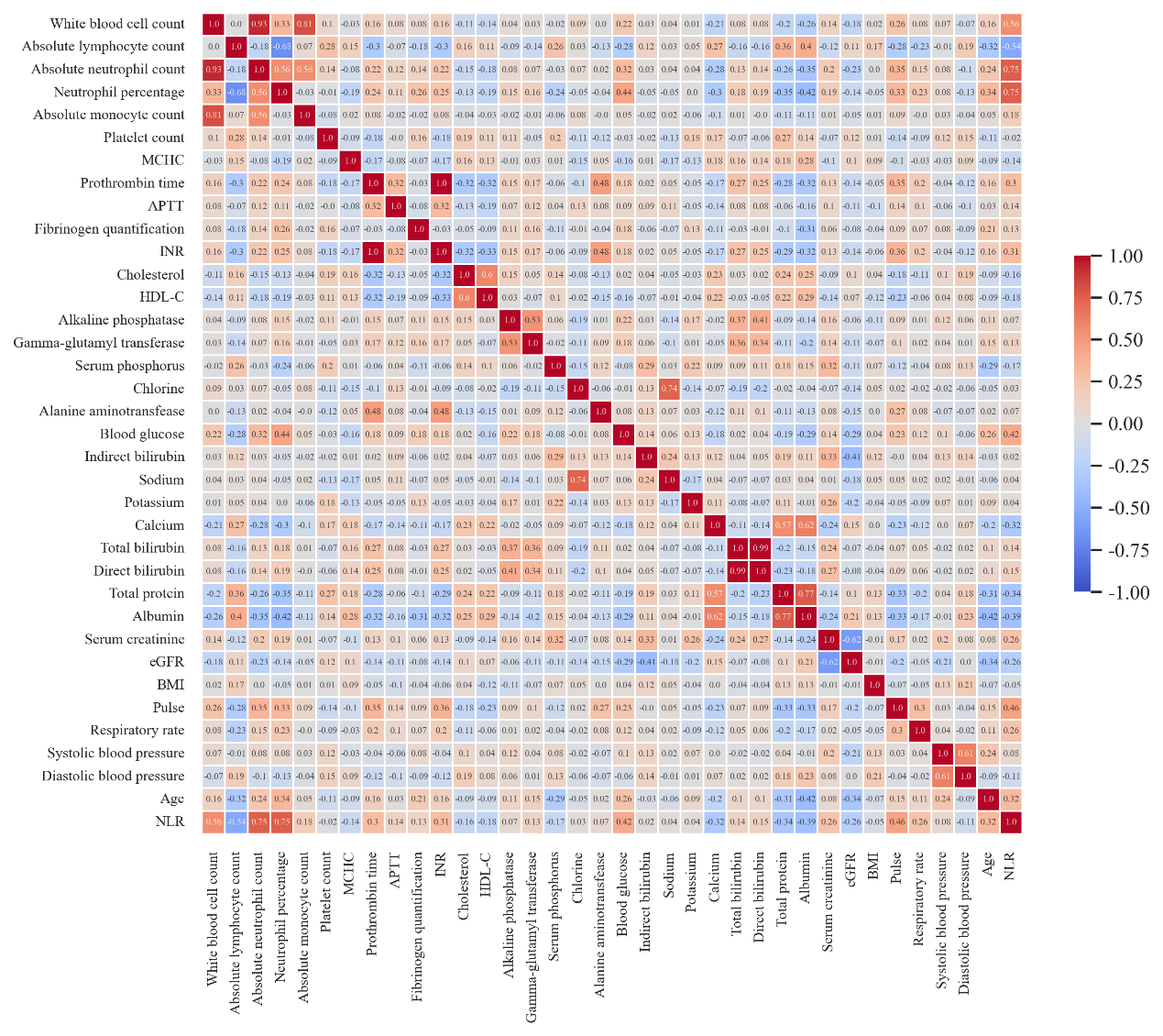
**

**Figure S2.** Correlation heat map between features


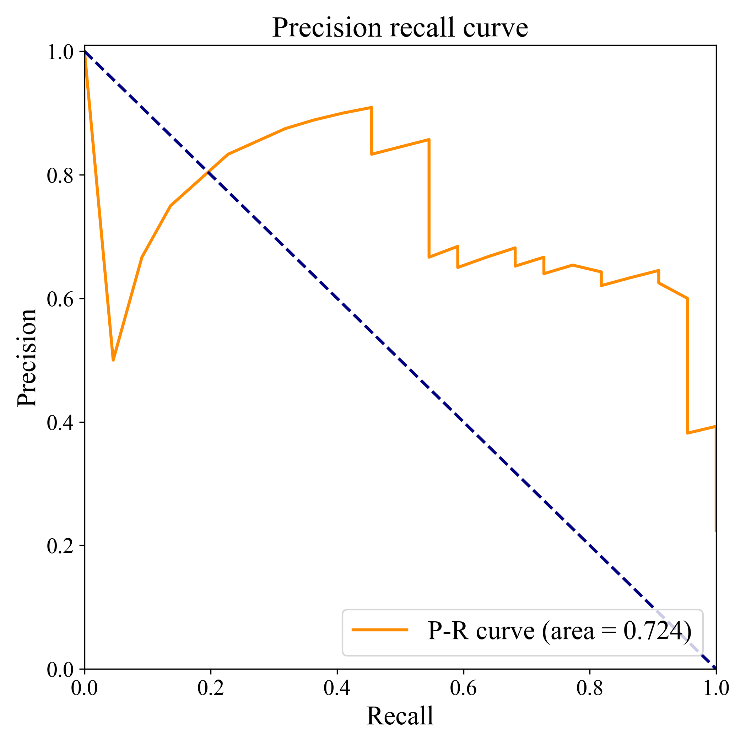


**Figure S3.** The Precision-Recall Curve in the internal validation of the RF model.


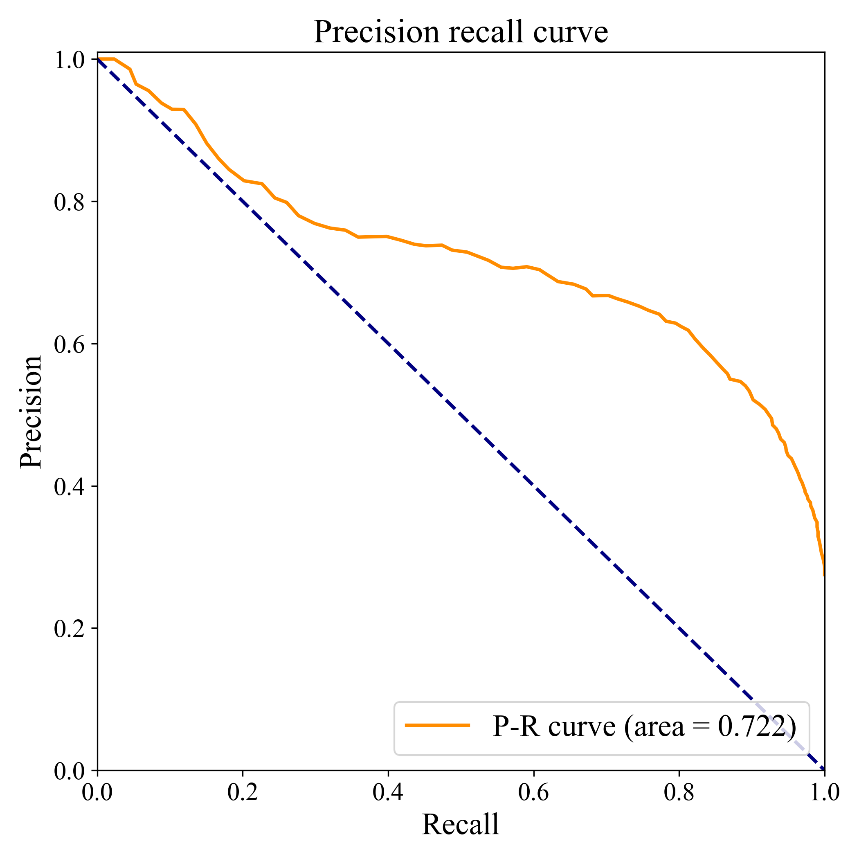


**Figure S4.** The Precision-Recall Curve in the external validation of the RF model.


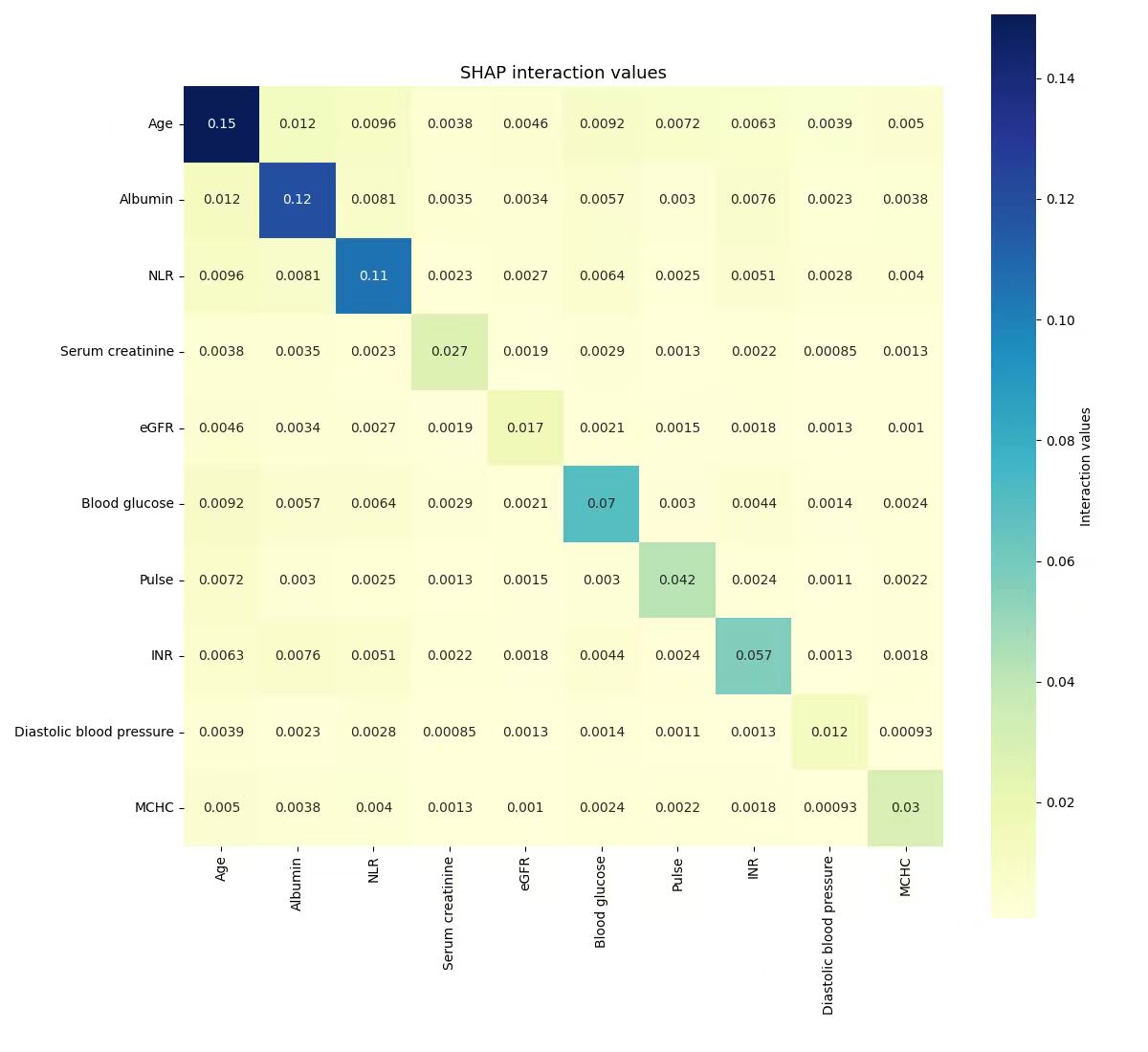


**Figure S5.** SHAP Interaction Value Matrix of Predictive Features for Postoperative Heart Failure
