# Supplementary material for: Development and validation of an interpretable machine learning model for predicting the risk of non-cardiac surgery postoperative heart failure: a multicenter study
Source: Front Med (Lausanne). 2025 Dec 11;12:1666885. doi: 10.3389/fmed.2025.1666885 (PMC12738863; doi:10.3389/fmed.2025.1666885)

Central illustration: Machine learning prediction postoperative heart failure for non-cardiac surgical patients

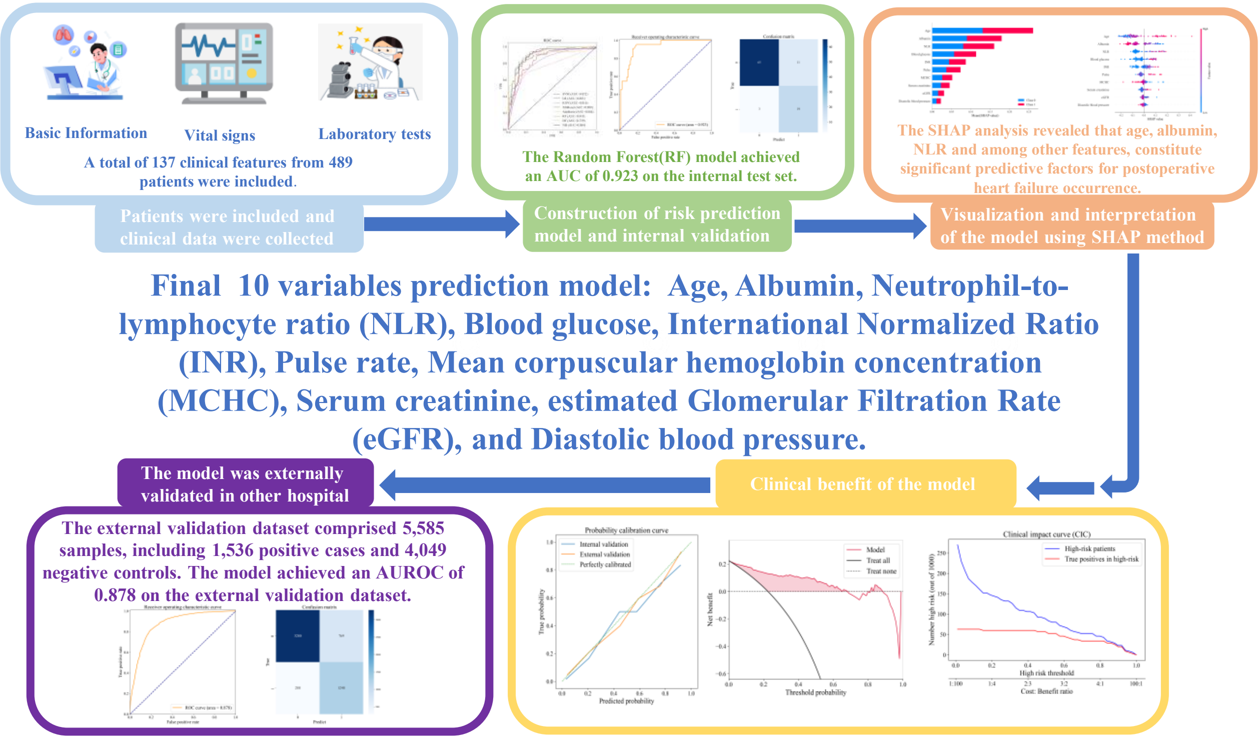

Supplement: Supplementary file 2 [file Data_Sheet_2.pdf]
